# Influence of load-bearing angle, structural topology, and porosity gradient on the energy absorption capability of TPMS-based scaffolds for bone tissue engineering

**DOI:** 10.3389/fbioe.2025.1638143

**Published:** 2025-09-02

**Authors:** Chunli Zhang, Yongtao Lyu, Anna Semenova, Zhonghai Li

**Affiliations:** ^1^ Senior Department of Orthopedics, The Fourth Medical Center, Chinese PLA General Hospital, Beijing, China; ^2^ Department of Engineering Mechanics, Dalian University of Technology, Dalian, China; ^3^ Department of Orthopedics, The first Affiliated Hospital, Dalian Medical University, Dalian, China

**Keywords:** TPMS scaffold, energy absorption performance, porosity gradient, load-bearing angle, structural design

## Abstract

**Introduction:**

Triply periodic minimal surfaces (TPMS) based scaffolds are widely used in bone tissue engineering for fixing bone fractures. The bone scaffolds implanted in the human body may receive shocking or impact loading during daily activities. However their energy absorption (EA) behaviors are still not systematically investigated in the literature.

**Methods:**

In the present study, the influence of load_bearing angle, the structural topology and porosity gradient on the EA behaviors of TPMS-based scaffolds are investigated. Uniform and porosity gradient Gyroid and IWP structures at the load-bearing angles of 0°, 15°, 30°, 45°, 60°, 75° and 90° were created. Mechanical testing and finite element analysis were performed to investigate their EA behaviors, which were characterized using the energy absorption efficiency (EAE), the specific energy absorption (SEA), the levels of the densification strain, plateau stress and stress distribution.

**Results:**

The results showed that the load-bearing angle plays an important role in EAE, SEA and plateau stress of porosity gradient Gyroid and IWP scaffolds. Different from the uniform TPMS scaffolds, the porosity gradient Gyroid and IWP scaffolds showed layer-to-layer damage behaviors, which reinforced their load-bearing capability and consequently the SEA performance is improved.

**Conclusion:**

The data in the present study provided important guidance on the selection and design of TPMS structures for bone tissue engineering applications.

## 1 Introduction

Bone fracture is a significant global health challenge, particularly affecting the elderly population (Wong et al., 2022). The number of orthopedic injuries is rising each year, and total hip arthroplasty (THA) has become one of the most common surgical procedures worldwide (Clar et al., 2024). Currently the allograft and autograft are two clinical methods for fixing the bone fractures. However, the allograft has the drawbacks of insufficient supplies and complications, and the autograft has the risks of disease transmission and genetic mismatches (Wisanuyotin et al., 2022). Due to these unavoidable limitations in both approaches, tissue engineering scaffolds have become a promising alternative for fixing bone fractures.

When designing bone scaffolds to replace the fractured bone tissues, the microstructure of the scaffolds plays a critical role, because it not only directly influences the mechanical behavior of the scaffolds, but also affects the cellular responses and tissue regeneration processes (Karageorgiou and Kaplan, 2005). Regarding the state-of-the-art on the development of the microstructure of bone scaffolds, three primary categories have emerged. The first is the one formed by regular and periodically arranged unit cells (Boccaccio et al., 2018; Rodríguez-Montaño et al., 2018). This type of scaffold was widely used in the early stage of bone tissue engineering. However, it faces limitations such as stress concentrations, poor cell affinity, etc. The second type is the one formed by the triply periodic minimal surfaces (TPMS). The TPMS structures exhibit a mean curvature of zero, which has been proved to enhance cell attachment (Vijayavenkataraman et al., 2018; Chen et al., 2019). Additionally, their high surface-to-volume ratio provides more space for bone regeneration, making the TPMS-based designs a popular choice for scaffold development (Jiang et al., 2024; Lai et al., 2025). The third type is the one formed by non-regular unit cell and spatially non-periodically arranged (Gómez et al., 2016; Wang et al., 2018). These include the Voronoi-based scaffolds, the Spinodoid-based scaffolds, etc. (Wang H. et al., 2025). While these scaffolds exhibit more biomimetic microstructures, their designs and high-quality manufacturing remain big challenges (Wang et al., 2018). It should be noted that in addition to the three main types mentioned above, there are the functionally graded porous scaffolds (Liu et al., 2018), the hybrid TPMS cellular scaffolds (Novak et al., 2021a; Zhang et al., 2025), functionally graded hybrid structures (Liu et al., 2018), etc. The experimental and numerical investigations showed that through novel design approach, such as hybrid or functionally graded strategies, excellent mechanical performance, such as superior strength, the break of the stiffness-tunability trade-offs, can be achieved. Therefore, the design of the microstructure of bone scaffolds is a crucial step and many factors, such as the structural topology, the porosity gradient direction, deserves to be investigated to increase the performance of the scaffolds.

Considering various factors such as stress concentration, cell attachment and the feasibility of design and manufacturing, TPMS-based scaffolds remain among the most widely adopted structural designs for bone tissue engineering. Recent advancements have further enhanced their functionalities. For instance, Liu et al. (2022) developed novel TPMS-based scaffolds with high porosity and improved mechanical properties, proposing a topology control strategy to address both mechanical and biological requirements. Through innovative design, Peng et al. (2023) obtained tunable mechanical and mass-transport properties of scaffolds by incorporating an inner pore within the TPMS structures. Experimental results demonstrated that the permeability and mechanical performance could be effectively decoupled, offering a promising foundation for the design of high-performance bone implants. In Liu et al. (2023) study, the fatigue behaviors of TPMS scaffolds were investigated and a strategy combining hot isostatic pressing and electropolishing was proposed to enhance the fatigue resistances of these scaffolds. To alleviate the stress shielding and improve the mass transport capacity of TPMS bone scaffolds, Jiang et al. (2024) and Lai et al. (2025) introduced multi-functional pores in the TPMS surfaces. Their results revealed that a significant reduction in the effective elastic modulus (from 5.01 GPa to 2.30 GPa), and a nearly six-fold increase in permeability (from 8.58 × 10^−9^ m^2^ to 5.14 × 10^−8^ m^2^) can be achieved through the multi-functional pores. However, it should be noted that the systematic investigation on the energy absorption behavior of TPMS scaffolds remains missing. When the TPMS-based intervertebral disc or the bone scaffolds are implanted into the human body, they may encounter a shock or impact loading during daily or sports activities of the human subject. Scaffolds with superior energy absorption capabilities can alleviate such loads, protecting surrounding tissues. Therefore, the energy absorption performance represents a critical design consideration for bone tissue engineering scaffolds. In the authors’ previous study (Lyu et al., 2024), the energy absorption performance of TPMS based structures at different load-bearing angles was systematically investigated, in which the Gyroid, Diamond and IWP structures at load-bearing angles of 0°, 15°, 30°, 45°, 60°, 75° and 90° were studied. However, it should be noted that the porosity gradient effect was not investigated in this previous study and furthermore, the previous study was designed for general engineering applications and special considerations for bone tissue engineering were not considered.

The aim of this study is to systematically investigate the influence of load-bearing angle, structural topology, and porosity gradient on the energy absorption performance of TPMS-based structures and thus to provide guidance on the selection and design of TPMS-based scaffolds in bone tissue engineering.

## 2 Materials and methods

### 2.1 Design of TPMS based bone scaffolds

In this study, two TPMS topologies were selected: Gyroid and IWP (I-graph and Wrapped Package-graph), to investigate the influence of structural topology on the energy absorption behavior of TPMS structures. The mathematical equations for these two topologies are as follows:

$$\text{Gyroid}:\sin\left(kx\right)\cos\left(ky\right)+\sin\left(ky\right)\cos\left(kz\right)+\text{si}n\left(kz\right)\cos\left(kx\right)=t$$ (1)

IWP:

$$2\left[\cos\left(kx\right)\cos\left(ky\right)+\cos\left(ky\right)\cos\left(kz\right)+\cos\left(kz\right)\cos\left(kx\right)\right]-\cos\left(2kx\right)-\cos\left(2ky\right)-\cos\left(2kz\right)=t$$ (2)

where *x*, *y* and *z* are the coordinates of a point in the design space; *k* defines the unit cell dimension in the *x*, *y,* and *z* directions; *t* linearly correlates with the volume fraction of the TPMS structures. For the porosity gradient structure, *t* is a function of the spatial coordinate. The gyroid and IWP structures can be defined by Equations 1, 2, respectively.

$$t=a_{0}+a_{1}x+a_{2}y+a_{3}z$$ (3)

where, $a_{0}$ , $a_{1}$ *,* $a_{2}$ and $a_{3}$ are the parameters to control the spatial variation in structural porosity. The structures with gradient porosities can be defined by Equation 3.

**FIGURE 1:**
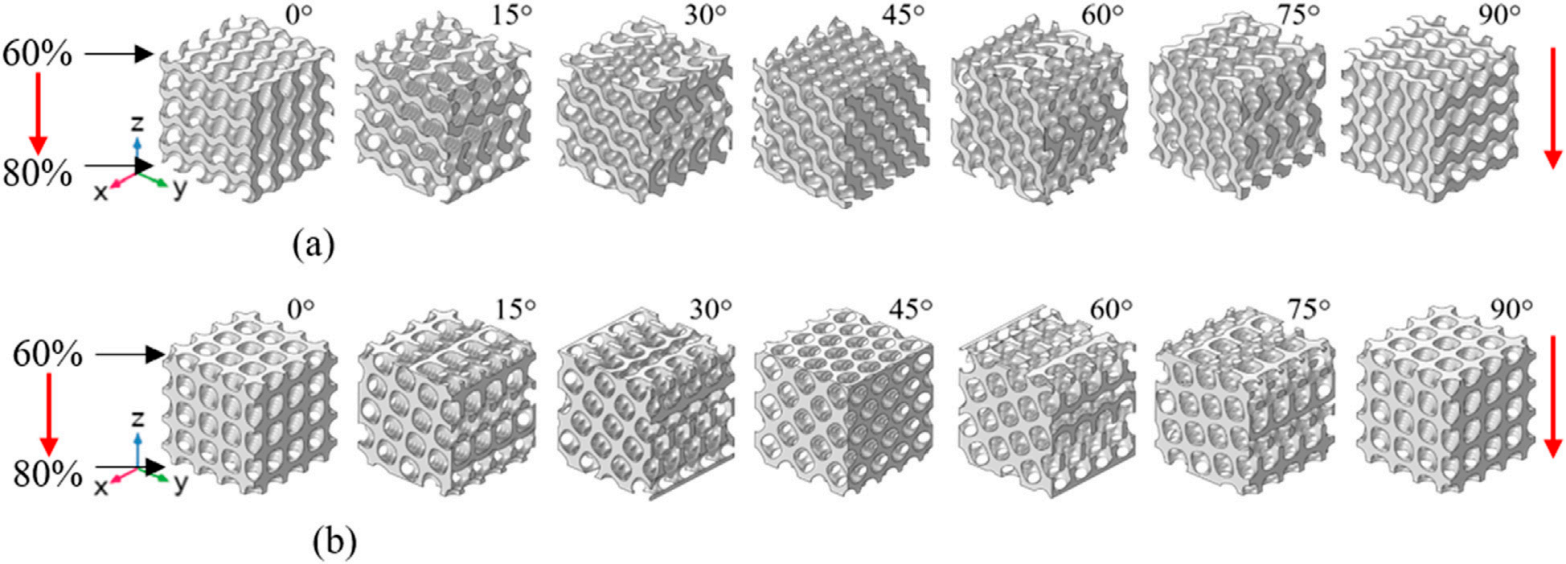
Illustration of the porosity gradient Gyroid and IWP structures at different load-bearing angles (the red arrows represent the loading and porosity gradient directions). **(a)** Gyroid. **(b)** IWP.

To investigate the influence of porosity gradient on the energy absorption behavior, both uniform and porosity-graded TPMS scaffolds were designed. For the uniform structures, porosity was fixed at 70%, while porosity gradient structures were designed with the porosity ranging from 60% to 80%, which meet the requirements in the bone tissue engineering applications (Karageorgiou and Kaplan, 2005). When designing the porosity gradient structures, the porosity at one end of the structures was set to 60% and linearly increased to 80% at the other end by controlling the parameter *t* in Equations 1, 2; Figure 1). Our previous study (Lyu et al., 2024) showed that the mechanical properties of 4 × 4 × 4 structures have reached the convergence, meaning the number of unit cell will not influence the results anymore, and thus the 4 × 4 × 4 structures were used throughout this study.

To analyze the influence of the load-bearing angle on the energy absorption behavior of TPMS-based scaffolds, the 4 × 4 × 4 Gyroid and IWP scaffolds were rotated along the *x*-axis by 0°, 15°, 30°, 45°, 60°, 75° and 90°, yielding seven different models per topology (Figure 1). When designing the rotated structures, the parameter *t* in Equations 1, 2 was set as a function of the *x*, *y* and *z* coordinates, and the parameters $a_{0}$ , $a_{1}$ *,* $a_{2}$ and $a_{3}$ in the function were properly controlled to ensure the porosity gradient direction is aligned with the loading direction, i.e., from top to bottom along *z*-axis in Figure 1. All the scaffolds were designed using the open-source software package–FLatt Pack (University of Nottingham, Nottingham, UK) (Maskery et al., 2022).

### 2.2 Additive manufacturing and quasi-static mechanical testing

**FIGURE 2:**
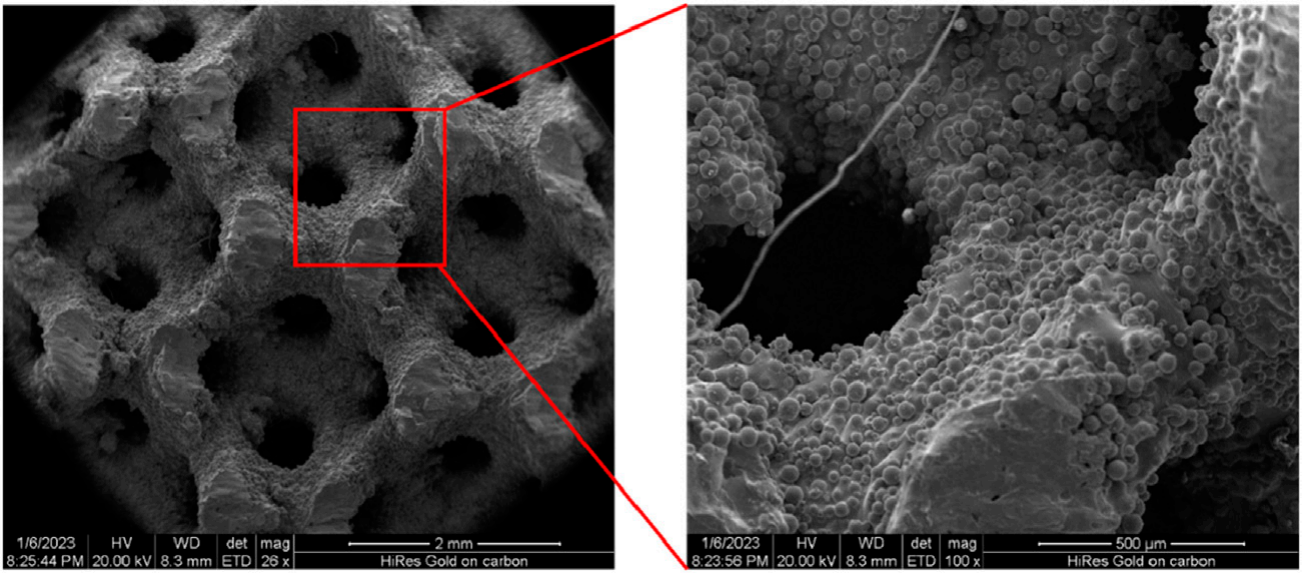
Scanning electron microscope (SEM) images of the scaffold made by the selective laser melting technique.

In the present study, the mechanical testing was one of the approaches used to investigate the energy absorption behavior of the scaffolds. Accordingly, the designed samples were fabricated with a dimension of 12.0 mm × 12.0 mm × 12.0 mm, with the size of each unit cell to be 3.0 mm × 3.0 mm × 3.0 mm. Given the complex microstructure of the TPMS samples, the additive manufacturing (AM) technique was selected as the method for the sample production, specifically the selective laser melting (SLM) technique was used, in which technique support structures were required for successful production of the designed TPMS scaffolds. The TPMS models were converted into 3D stereolithography (STL) files and then imported into Materialise Magics (v12.0, Materialise NV, Leuven, Belgium) for the generation of supports prior to 3D printing. The specimens were manufactured using Ti6Al4V powder and using the 3D printing machine - Renishaw AM400 (Wotton-under-Edge, UK). Due to the high melting point of Ti6Al4V materials in the SLM process (Soro et al., 2019), the input laser power was set to 280 W and the scanning speed was set to 7.3 mm/s. After the 3D printing process, the samples were post-processed following the standard procedure reported in the literature (Gandhi et al., 2024; Le et al., 2025), i.e., the samples were placed at the temperature of 800 °C for 2 h to eliminate the residual stress, the micro-shot peening with corundum was used to remove the unmelted particles and to enhance the surface roughness, etc. The sample size was chosen to be six and thus six samples were produced for each design. To assess the quality of the AM produced samples, scanning electron microscope (SEM) imaging of the samples was performed and examples of the images are presented in Figure 2, showing that a good quality has been achieved, i.e., the curved shape has been preserved and no holes/voids in the struts are present, etc.

The fabricated samples were then subjected to the mechanical testing using a SANS compression testing machine, capable of delivering a maximum load of 50.0 kN, which is sufficient to fracture the samples. The quasi-static uniaxial compression testing was performed by applying a vertical compression load at the loading rate of 0.5 mm/min. In the test setup, two flat platens were positioned above and below the specimens, with the specimens centered on the platform. The force and displacement sensors were connected to the upper and lower platens to collect the reaction forces and displacement data from the experiment during the compression process. The acquired force-displacement data were subsequently converted to the stress-strain data, which were used to calculate the energy absorption properties of the TPMS scaffolds. For each design, the quasi-static uniaxial compression testing was repeated on the six samples and the mean and standard deviation (SD) of the experimental data were obtained for the subsequent analysis.

### 2.3 Finite element analysis of the bone scaffolds

In the present study, the finite element method (FEM) is another method used to investigate the energy absorption behavior (especially the stress distribution within the structure) of the TPMS-based bone scaffolds. To generate the finite element (FE) models, the designed TPMS geometric models were imported into HyperMesh (v10.0, Altair Engineering Inc., Troy, MI, USA), where they were meshed using the second order tetrahedron element (C3D8). A mesh convergence study was performed to ensure that the FE results are independent of the mesh size. The mechanical properties of Ti6Al4V material were defined including an elastic modulus of 110.0 GPa, a Poisson’s ratio of 0.3, a Johnson-Cook plastic model with the yield stress of 850.0 MPa, the hardening modulus of 793.0 MPa and the hardening index of 0.386 (Ducobu et al., 2017). The scenario of quasi-static uniaxial compression test was simulated and thus two rigid plates were added to the upper and lower surfaces of the 4 × 4 × 4 FE models. Then the bottom rigid plate was fully constrained, and a displacement of 7.2 mm was applied on the top plate to ensure a 60% strain was achieved. In the FE simulation, for every finite element, when the failure point was reached, it was removed from the analysis and thus not involved in the subsequent analysis. The FE simulation was performed in Abaqus (v6.14, Dassault Systems SIMULIA Ltd, Providence, RI, UAS).

### 2.4 Energy absorption behaviors of the bone scaffolds

**FIGURE 3:**
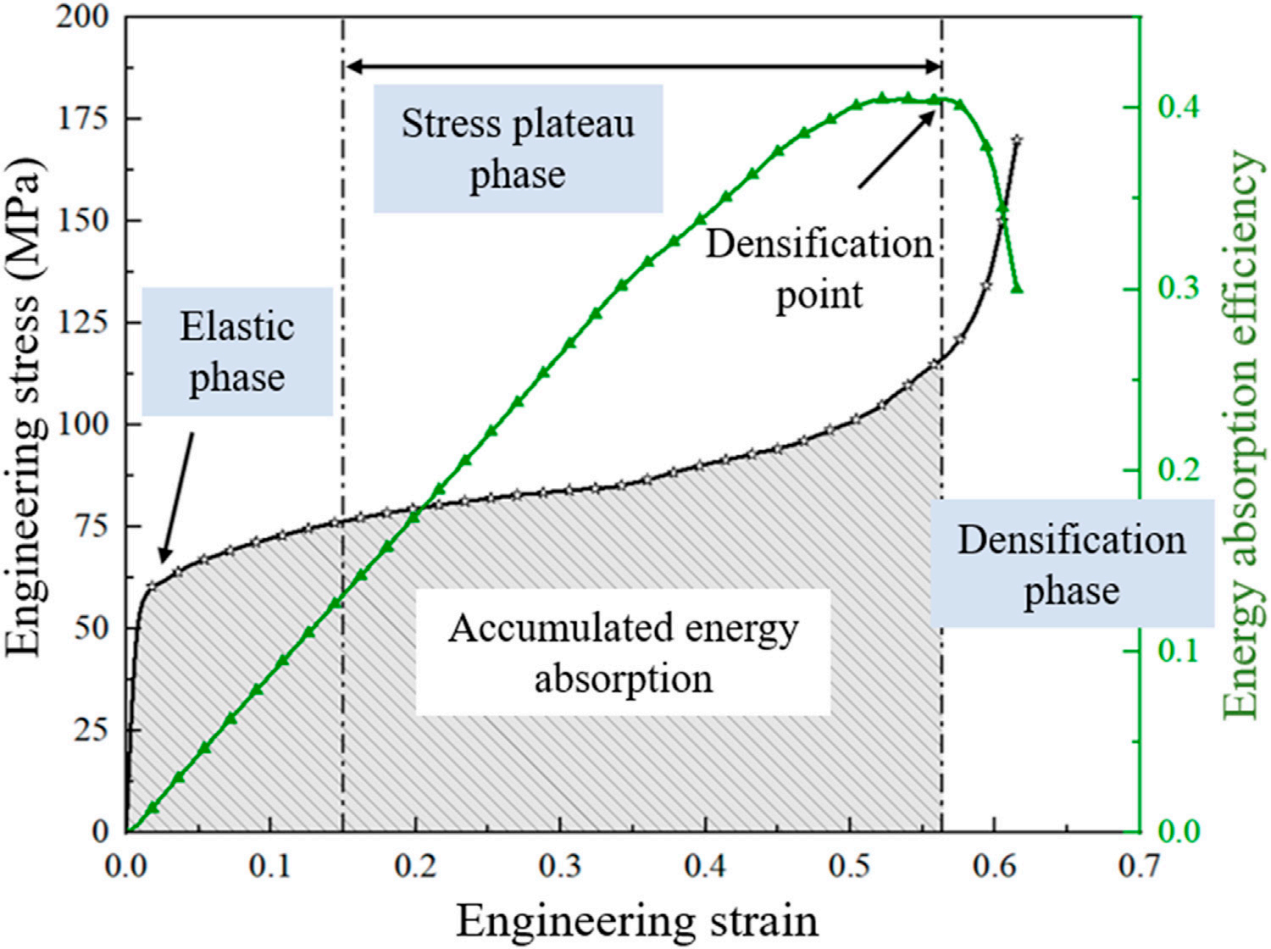
Illustration of the structural energy absorption related parameters.

In the present study, the energy absorption behaviors of the TPMS scaffolds were quantified using the energy absorption efficiency (EAE), the specific energy absorption (SEA), the levels of the densification strain and plateau stress (Figure 3). The energy absorption efficiency is defined as a function of the structural strain formulated as follows:

$$\text{EAE}=\frac{w}{\sigma \left(\varepsilon \right)}$$ (4)

where *w* represents the total energy absorbed by the structure during the compression process, which is given by $w=\int_{0}^{\varepsilon }\sigma \left(\varepsilon \right)d\varepsilon$ ; $\sigma \left(\varepsilon \right)$ is the structural stress during the compression process.

When the TPMS scaffolds are compressed to complete failure, their stress-strain curves can be categorized into three phases: the elastic phase, the stress plateau phase and the densification phase (Figure 3) (Qiu et al., 2024). The stress plateau phase is characterized by the stabilization of the stress value. The internal stress at this phase is defined as the plateau stress and can be expressed using the formulation below:

$$\sigma_{pl}=\frac{\int_{\varepsilon_{s}}^{\varepsilon_{e}}\sigma \left(\varepsilon \right)d\varepsilon }{\varepsilon_{e}-\varepsilon_{s}}$$ (5)

where $\sigma_{pl}$ is the plateau stress; $\varepsilon_{s}$ is the initial strain at the beginning of the plateau phase, at which point, the material yielding occurs; $\varepsilon_{e}$ is the strain at the end of the plateau phase, also called the densification strain, at which point, the corresponding stress is the ultimate strength of the material.

The energy absorption capability of the TPMS scaffold was quantified using the specific energy absorption (SEA), the formulation of which is given as below:

$$\text{SEA}=\frac{w_{v}}{M}$$ (6)

where *M* (unit in kg/m3) denotes the mass of the structure per unit volume; $w_{v}$ (unit in kJ/m3) represents the total energy absorbed by unit volume stress when the strain reaches the densification strain. Therefore, the energy absorption capacity can be characterized by Equations 4-6.

## 3 Results

### 3.1 Influence of load-bearing angle, topology and porosity on the SEA and EAE of TPMS scaffolds

**FIGURE 4:**
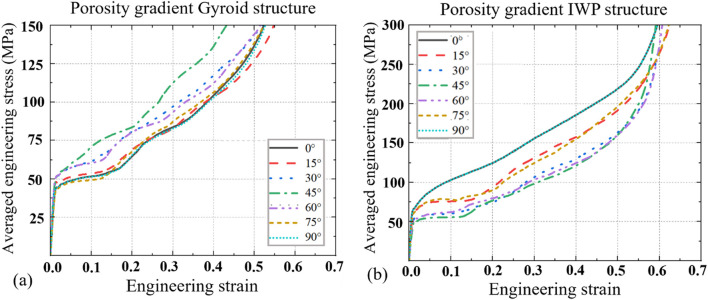
The averaged engineering stress *versus* engineering strain curves for the porosity gradient Gyroid and IWP structures at different load-bearing angles (*n* = 6). **(a)** Gyroid. **(b)** IWP.

**FIGURE 5:**
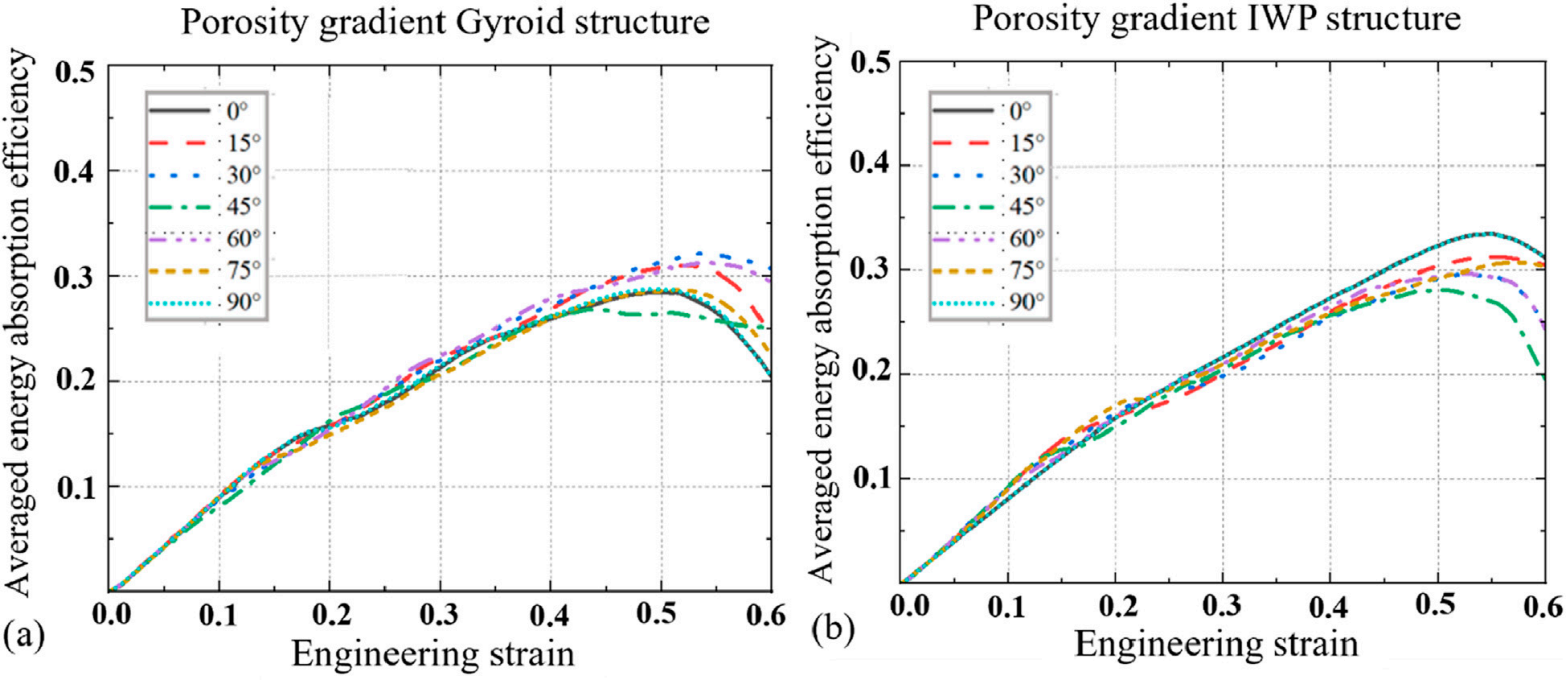
The averaged Energy absorption efficiency for the porosity gradient Gyroid and IWP structures at different load-bearing angles (*n* = 6). **(a)** Gyroid. **(b)** IWP.

**FIGURE 6:**
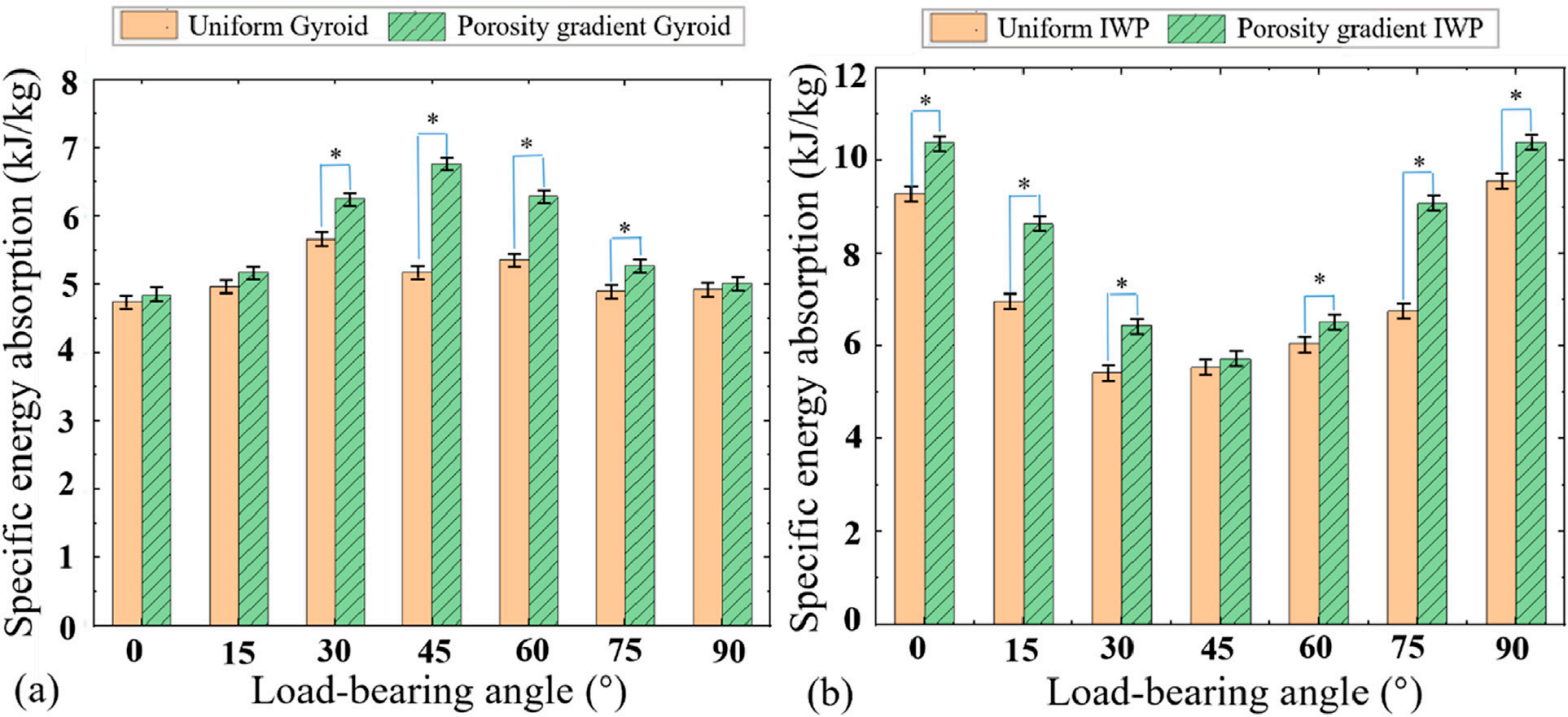
Comparisons of the specific energy absorptions between the uniform and porosity gradient Gyroid and IWP structures at different load-bearing angles (*n* = 6, *p < 0.05). **(a)** Gyroid. **(b)** IWP.

The stress-strain curves of the porosity gradient Gyroid and IWP scaffolds obtained at different load-bearing angles are shown in Figure 4 and the corresponding SEA and EAE values are shown in Figures 5, 6. Key findings include: (1) Comparing the porosity gradient Gyroid-based structures with the porosity gradient IWP-based scaffolds, the influence of loading-bearing angle on the stress-strain curves is different. For the porosity gradient Gyroid scaffolds, the structure at 45° exhibits the highest SEA, while for the porosity gradient IWP, the structures achieved peak SEA at 0° and 90°. Interestingly, the uniform Gyroid and IWP structures exhibit the same phenomenon (the reader can refer to the authors’ previous publication (Lyu et al., 2024) for the stress-strain curves of uniform counterparts); (2) The Energy absorption efficiency (EAE) of porosity gradient Gyroid and IWP scaffolds are at the similar level but the influence of the load-bearing angle on the EAE is different. For the porosity gradient Gyroid structure, the highest efficiency occurs at 30°, whereas the porosity gradient IWP scaffolds showed the peak efficiency at 0° and 90°; (3) The t-test analysis was performed to statistically analyze the differences observed in SEA between the uniform and porosity gradient Gyroid and IWP structures (Figure 6). Compared to the uniform counterparts, the porosity gradient Gyroid structures exhibited statistically higher SEA at the load-bearing angles of 30°, 45°,60° and 75° (*p* < 0.05), and the porosity gradient IWP structures exhibited statistically higher SEA at the load-bearing angles of 0°, 15°, 30°, 60°, 75°and 90° (*p* < 0.05).

### 3.2 Influence of load-bearing angle and topology on the densification strain and stress distribution of TPMS scaffolds

**FIGURE 7:**
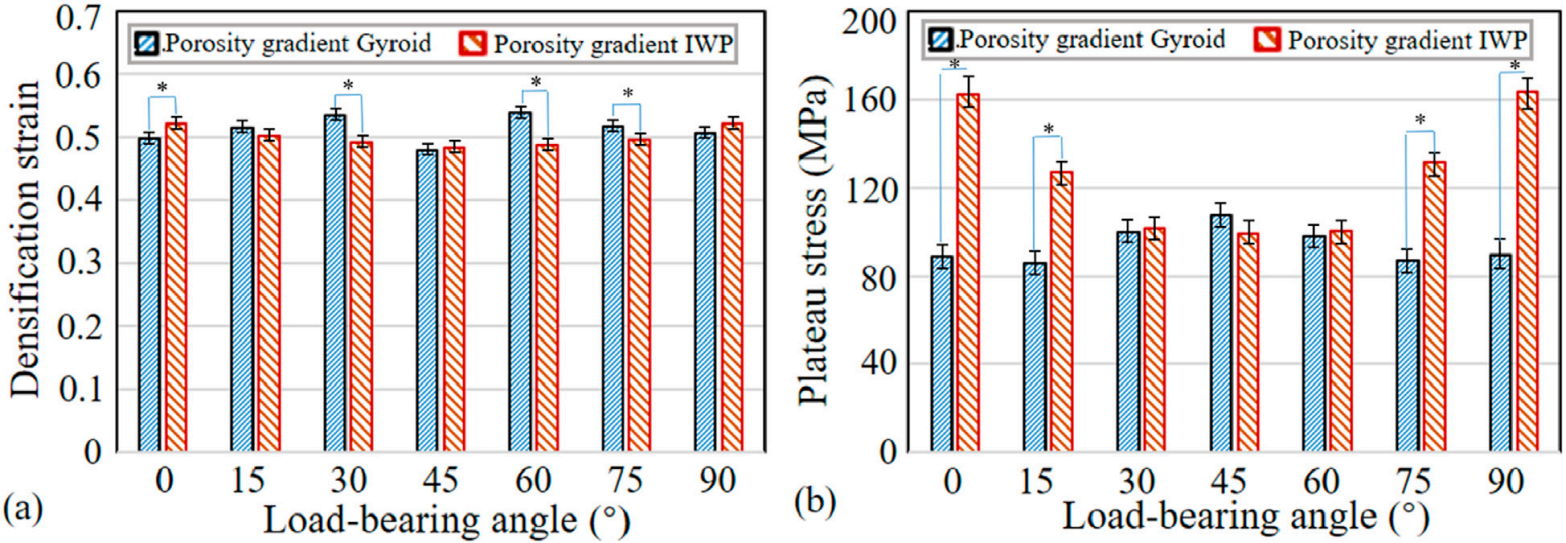
Comparisons of the densification strain and plateau stress between the porosity gradient Gyroid and IWP structures at different load-bearing angles (*n* = 6, *p < 0.05). **(a)** Gyroid. **(b)** IWP.

The densification strains and plateau stresses across various load-bearing angles are shown in Figure 7. The t-test analysis was performed to statistically analyze the differences between the porosity gradient Gyroid and porosity gradient IWP structures. Key observations include: (1) Compared to the porosity gradient IWP structures, the porosity gradient Gyroid scaffolds exhibited statistically higher densification strains at 30°, 60° and 75° (*p* < 0.05), while at the load-bearing angle of 0°, the porosity gradient Gyroid-based scaffolds exhibited statistically lower densification strain; (2) Compared to the porosity gradient Gyroid structures, the porosity gradient IWP structures exhibited statistically higher plateau stresses at the load-bearing angle of 0°, 15°, 75° and 90° (*p* < 0.05), while at other load-bearing angles, there is no statistical difference in the plateau stress between the two types of structures.

**FIGURE 8:**
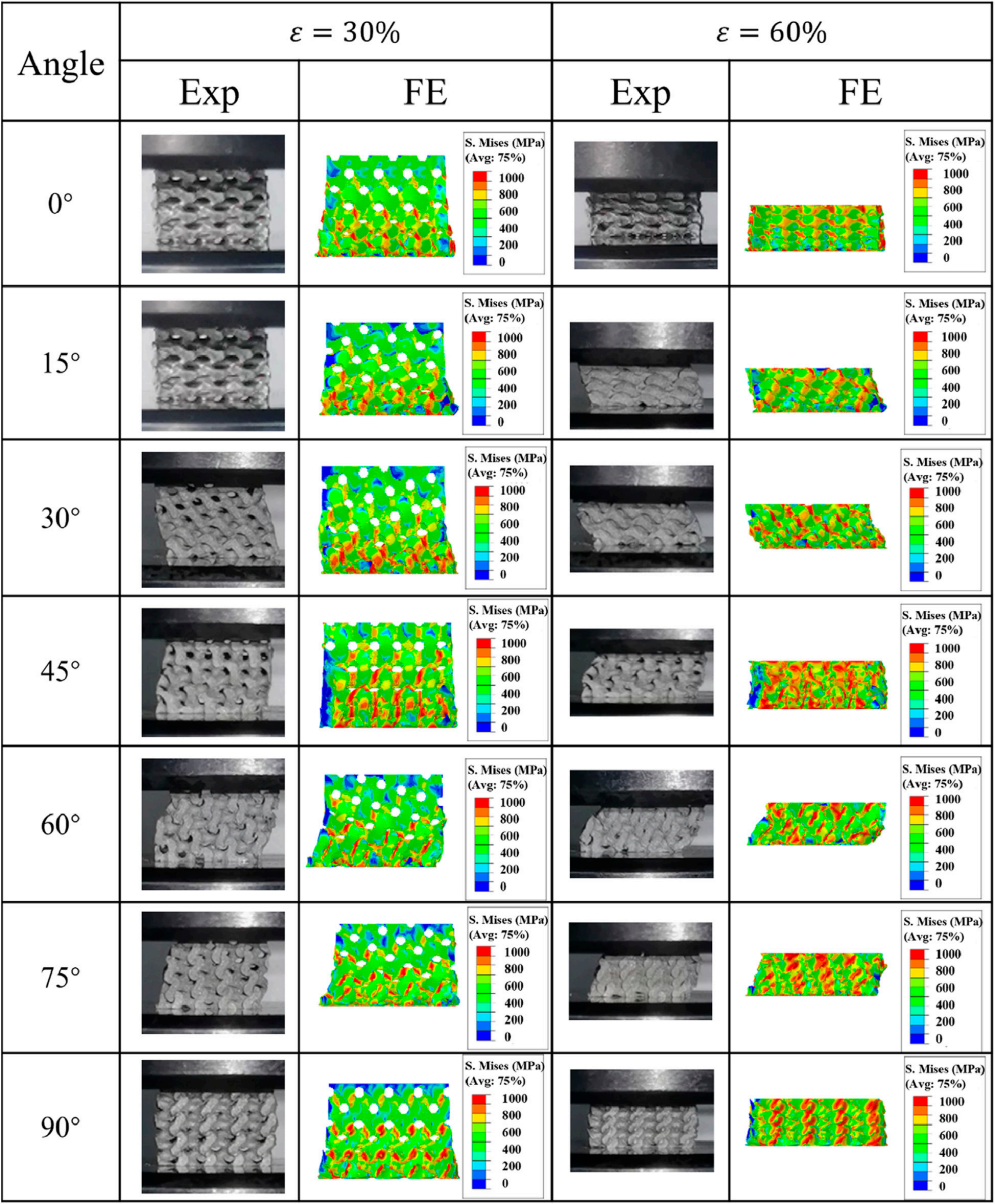
Comparison of the von Mises stress distribution in the porosity gradient Gyroid structures at different load-bearing angles.

**FIGURE 9:**
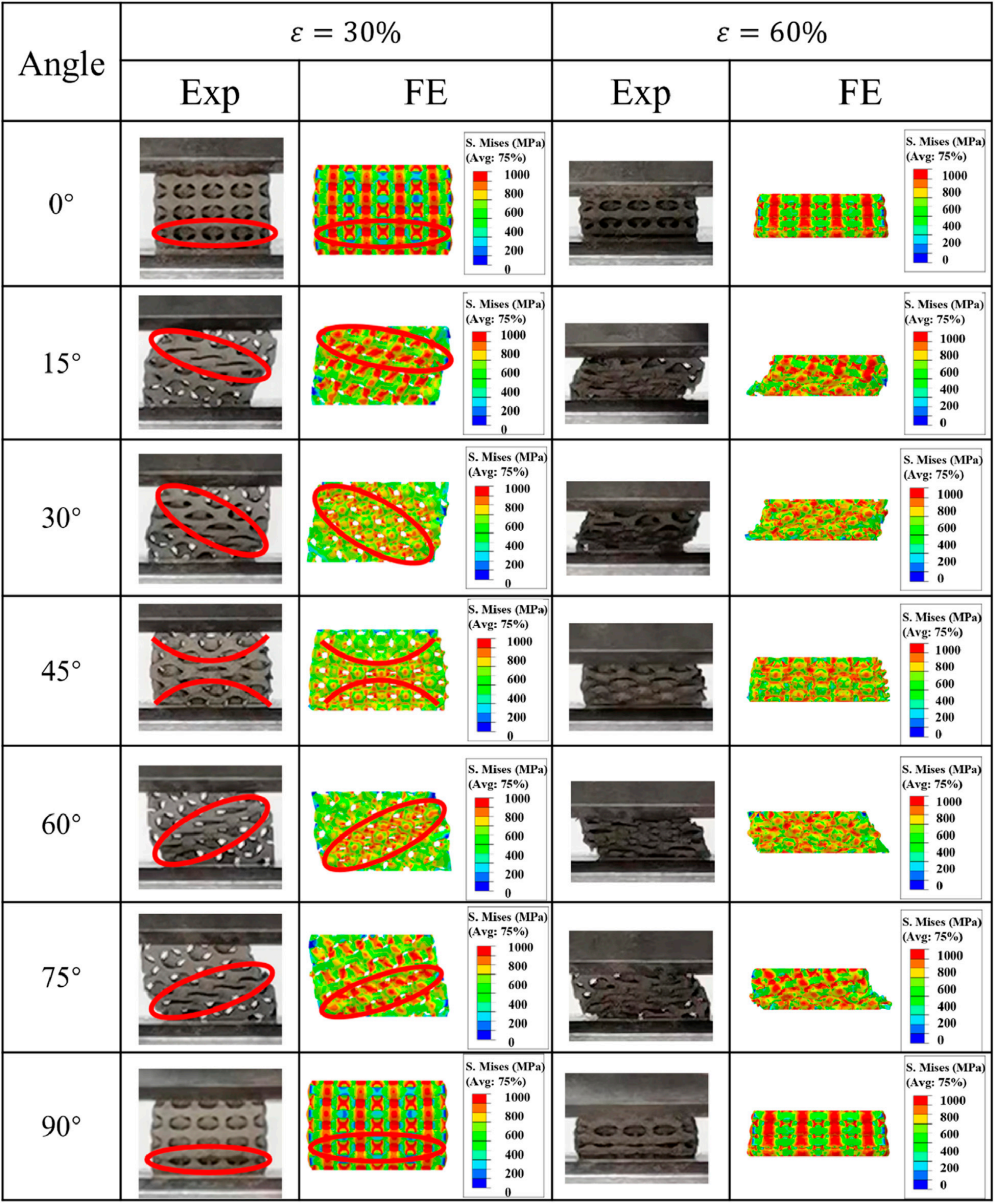
Comparison of the von Mises stress distribution in the porosity gradient IWP structures at different load-bearing angles.

The stress distributions inside the porosity gradient Gyroid and IWP structures during the uniaxial compression process are illustrated in Figures 8, 9. It can be seen for both Gyroid and IWP structures, the stress distribution inside the structure is not uniform in the compression process. The high stress always occurred in the high porosity side and then propagated to the low porosity side. As a result, the structural damage occurred in the high porosity side first and then propagated layer by layer to the low porosity side (upper side). Therefore, the yield stress of the gradient structure depends on the load-bearing capability of the high porosity part. This phenomenon is true for both Gyroid and IWP structures. It should be noted that although the porosity gradient structure is more prone to yield compared to the uniform structure, a layer-by-layer damage is induced in the porosity gradient structure, and consequently the plateau stress was increased and thus the energy absorption capability was increased.

## 4 Discussion

In this paper, the influence of load-bearing angle, the structural topology, and the porosity gradient on the energy absorption behaviors of TPMS-based scaffolds was investigated. Uniform and porosity gradient Gyroid and IWP scaffolds at load-bearing angles of 0°, 15°, 30°, 45°, 60°, 75° and 90° were created. Finite element analysis and mechanical testing were used to investigate their energy absorption behaviors. The following two interesting findings are revealed from the present study. It should be noted that different from our previous publication (Lyu et al., 2024), in which only the uniform TPMS structures were investigated, the present study focuses more on the mechanical behaviors of the porosity gradient TPMS structures and therefore, some new findings and insights were drawn from this study.

First, the load-bearing angle significantly influences the energy absorption performance of the porosity gradient TPMS-based structures. Previous study has shown that the TPMS scaffolds exhibits an anisotropic elastic behavior, characterized by Zener anisotropy factor deviating from 1.0 (Lu et al., 2019). This study further reveals that the structural anisotropy can further influence both the SEA and plateau stress in both uniform and porosity gradient TPMS scaffolds. Regarding the SEA, it should be noted that the influence of load-bearing angle exhibits different scenarios for porosity gradient Gyroid and IWP-based scaffolds: while the porosity gradient Gyroid exhibits the highest SEA at 45°, the porosity gradient IWP exhibits the highest SEA at 0° and 90°. This highlights the necessity of evaluating the effects of load-bearing angle individually for each topology in practical applications. Strategic adjustment of load-bearing angle can thus improve the SEA of porosity gradient TPMS-based scaffolds. Regarding the plateau stress, the influence of load-bearing angle also differs for porosity gradient Gyroid and IWP-based scaffolds: The porosity gradient IWP exhibits high plateau stresses at 0° and 90°, while the porosity gradient Gyroid exhibits almost similar plateau stress at different load-bearing angles, the reason of which could be that they possess different structure characteristics. As shown in Figure 1, the struts of porosity gradient IWP are aligned with the loading direction at 0° and 90° and thus the bearing loads are high and consequently the plateau stress is high, while the porosity gradient Gyroid possesses curved struts and thus the bearing loads are similar at different angles. In contrast, the densification strains of porosity gradient Gyroid and IWP-based scaffold are not significantly influenced by the load-bearing angle. The reason could be that the densification strain reflects the duration period of the plateau stress, and it is the point when the porous structures change from porous to dense state (Singh et al., 2024). Therefore, the densification strain is more related to the porosity of the structures. In this paper, since all the tested structures shared similar porosity levels, their densification strains remained comparable.

Second, compared to the uniform TPMS structures, the porosity gradient TPMS structures (both Gyroid and IWP) exhibit enhanced energy absorption performance. As shown in Figure 3, superior energy absorption is characterized by a larger area under stress-strain curve (i.e., the shaded region), which represents cumulative energy absorption. This improvement can be achieved through two mechanisms: elevating plateau stress and extending the duration of the plateau stress plane. In this study, the data showed that both elevated plateau stress and prolonged plateau stress period are achieved in the porosity gradient TPMS structures (compared to the uniform counterparts). These findings are in the agreement with the data published in the literature (Song et al., 2024; Wang Z. et al., 2025). Therefore, this study further confirms that: the TPMS structure can absorb more energies by defining different porosities in different parts of the structure; Additionally, the porosity gradient TPMS structure exhibit a layer-to-layer damage behavior, and consequently the stress concentration can be eased. As a result, under the impact loading, the porosity gradient structure can better dissipate the stress and absorb more energies. Therefore, this study suggests that the porosity gradient TPMS structures (rather than the uniform porosity ones) should be used in bone scaffolds for the purpose to protect the human body during everyday activities, especially in the impact and shock loading scenarios.

This study has several limitations that warrant discussion, along with opportunities for further research. First, there may be some manufacturing errors in the produced samples and the influence of manufacturing errors on the results was not addressed in the present study. In the authors’ previous study (Lu et al., 2020), microCT imaging analysis has been performed to quantify the discrepancies in the geometric properties of TPMS scaffold, SEM imaging has been used to inspect the surface roughness of the produced samples, FE modeling and experimental testing have been used to quantify the discrepancies in the mechanical properties of the theoretically designed and additively manufactured scaffolds. In the present study, although the SEM images showed a good quality of the samples, the discrepancies in the geometric and mechanical properties of TPMS scaffolds still need to be quantified using microCT scanning, FE modeling, experimental testing, and the influence of these discrepancies on the energy absorption behavior of the TPMS scaffolds needs to be further investigated in the future. Second, the energy absorption behavior of the scaffolds was investigated only under the quasi-static loading scenario. The scaffolds may be subjected to different types of loading, including the shock and dynamic loading. Previous studies (Feng et al., 2023; Li et al., 2024; Novak et al., 2021b) have shown that the strength of the TPMS-based structures under dynamic loading was apparently higher than the static results, indicating TPMS structures present certain strain rate sensitivity, additionally they found that the TPMS-based structures possess good potential for use in crashworthiness applications, etc. Therefore, more loading scenarios should be generated to systematically investigate the dynamic behaviors of TPMS-based structures in the future. Last but not the least, only the behavior of the 4 × 4 × 4 structure was investigated. In the future, the energy absorption behavior of the TPMS based clinical products should be investigated, such as the TPMS based artificial intervertebral disc and the TPMS based hip stem, etc. In this way, the influence of the irregular outer shape on the mechanical behavior can be considered and the results will be more clinically relevant. Additionally, since there is still no consensus on the post-processing of the 3D printed Ti6Al4V TPMS scaffolds (Gandhi et al., 2024; Le et al., 2025), the influence of the different post-processing procedures on the mechanical behaviors of TPMS scaffolds needed to be systematically investigated in the future.

## 5 Conclusion

In this study, the energy absorption behaviors of the uniform and porosity gradient Gyroid and IWP structures at the load-bearing angles of 0°, 15°, 30°, 45°, 60°, 75° and 90° were investigated. The following conclusions can be drawn.

1. The load-bearing angle significantly influences the energy absorption performance (including the SEA, EAE, plateau stress, etc.) of the porosity gradient TPMS structures. Therefore, when designing TPMS-based scaffolds, the load-bearing angle of the scaffold should be one important design variable to be considered.
2. The porosity gradient TPMS structures (both Gyroid and IWP) exhibited layer-to-layer damage behavior, which increased their loading capability, elevated the plateau stress and thus their energy absorption performance (e.g., SEA) was improved compared to their uniform counterparts. Therefore, when designing TPMS-based bone scaffolds, the porosity gradient of the scaffold can be utilized to increase the shock-resistant and impact-resistant properties of the scaffolds.

The data presented in the present study provide important information for the selection and design of TPMS structures for bone tissue engineering applications.

## Data Availability

The original contributions presented in the study are included in the article/supplementary material, further inquiries can be directed to the corresponding authors.
